# Enhanced cardiac fatty acid utilization induced by high dietary fat: a potential regulatory role for mitochondrial aconitase

**DOI:** 10.1186/1753-6561-6-S3-P15

**Published:** 2012-06-01

**Authors:** Jolyn Fernandes, Luke I Szweda, Michael Kinter

**Affiliations:** 1 Free Radical Biology and Aging Program, Oklahoma Medical Research Foundation, Oklahoma City, OK 73104, USA; 2 Department of Biochemistry and Molecular Biology, University of Oklahoma Health Science Center, Oklahoma City, OK 73104, USA; 3 Department of Geriatric Medicine, Reynolds Oklahoma Center on Aging, University of Oklahoma Health Science Center, Oklahoma City, OK 73104, USA; 4 Department of Medicine, Section of Endocrinology and Diabetes, University of Oklahoma Health Science Center, Oklahoma City, OK73104, USA

## Background

Obesity is an independent risk factor for cardiovascular disease and Type 2 diabetes, a condition that enhances the risk for various cardiomyopathies. Obesity is known to induce a state of metabolic inflexibility within the heart characterized by increased mitochondrial reliance on fatty acids for energy production. While the molecular mechanisms are not fully clarified, these changes in metabolism are thought to contribute to degeneration of cardiovascular function. The focus of my research is to investigate regulatory mechanisms that control diet-induced changes in fatty acid utilization (β-oxidation) by cardiac mitochondria.

## Materials and methods

In the current study, we used quantitative proteomics, mitochondrial respiratory analysis, and enzymology to investigate factors that contribute to diet-induced changes in fatty acid utilization by cardiac mitochondria (β-oxidation). We employed C57B/6J mice fed a high fat diet (60% kcal from fat) as a model of obesity with mice fed low a fat diet (10% kcal from fat) serving as controls.

## Results

Whole pathway analysis by mass spectrometry revealed elevated expression of β-oxidation enzymes with no change in Krebs cycle enzymes within 2 wk on the high fat diet up to ≤ 60 wk. These observations indicate a mismatch between β-oxidation and the Krebs cycle that is responsible for the consumption of β-oxidation product, acetyl CoA. This could lead to the buildup of cardiotoxic fatty acid derived intermediates. Subsequent, enzymatic analysis revealed that citrate synthase, isocitrate dehydrogenase, and α-ketoglutarate dehydrogenase, the key regulatory enzymes of the Krebs cycle, exhibited no change in activity. However, mitochondrial aconitase activity was increased approximately 60% with no change in expression suggesting a role for post translational modifications. Increased aconitase specific activity was observed within 2 wk on the high-fat diet and persisted with extended dietary durations (≤ 60 wk).

## Conclusions

The increase in cardiac mitochondrial aconitase activity in response to high dietary fat highlights a potential novel regulatory role for the enzyme in β-oxidation. In the cytosol, citrate can be converted to malonyl CoA, a potent inhibitor of β-oxidation. Citrate is also an allosteric activator of acetyl CoA carboxylase, the enzyme that produces malonyl CoA. Since aconitase converts citrate to isocitrate, an increase in aconitase activity within the mitochondria has the potential to reduce citrate efflux and thus promote β-oxidation. These events could contribute to dietary-induced metabolic inflexibility.

## Funding

NIH.

